# Eliciting patient preferences and predicting behaviour using Inverse Reinforcement Learning for telehealth use in outpatient clinics

**DOI:** 10.3389/fdgth.2024.1384248

**Published:** 2024-10-31

**Authors:** Aaron J. Snoswell, Centaine L. Snoswell, Nan Ye

**Affiliations:** ^1^ Australian Research Council Centre of Excellence for Automated Decision Making and Society, Queensland University of Technology, Brisbane, QLD, Australia; ^2^ School of Information Technology and Electrical Engineering, University of Queensland, Brisbane, QLD, Australia; ^3^ School of Mathematics and Physics, University of Queensland, Brisbane, QLD, Australia; ^4^ Digital Media Research Centre, Queensland University of Technology, Brisbane, QLD, Australia; ^5^ GenAI Lab, Queensland University of Technology, Brisbane, QLD, Australia; ^6^ School of Communication, Queensland University of Technology, Brisbane, QLD, Australia; ^7^ Centre for Online Health, The University of Queensland, Brisbane, QLD, Australia; ^8^ Centre for Health Services Research, The University of Queensland, Brisbane, QLD, Australia

**Keywords:** Inverse Reinforcement Learning, machine learning, stated preference modelling, telehealth, telemedicine, behaviour modelling

## Abstract

**Introduction:**

Non-attendance (NA) causes additional burden on the outpatient services due to clinician time and other resources being wasted, and it lengthens wait lists for patients. Telehealth, the delivery of health services remotely using digital technologies, is one promising approach to accommodate patient needs while offering more flexibility in outpatient services. However, there is limited evidence about whether offering telehealth consults as an option can change NA rates, or about the preferences of hospital outpatients for telehealth compared to in-person consults. We model patient preferences with a Maximum Entropy Inverse Reinforcement Learning (IRL) behaviour model, allowing for the calculation of general population- and demographic-specific relative preferences for consult modality. The aim of this research is to use real-world data to model patient preferences for consult modality using Maximum Entropy IRL behaviour model.

**Methods:**

Retrospective data were collected from an immunology outpatient clinic associated with a large metropolitan hospital in Brisbane, Australia. We used IRL with the Maximum Entropy behaviour model to learn outpatient preferences for appointment modality (telehealth or in-person) and to derive demographic predictors of attendance or NA. IRL models patients as decision making agents interacting sequentially over multiple time-steps, allowing for present actions to impact future outcomes, unlike previous models applied in this domain.

**Results:**

We found statistically significant (*α* = 0.05) within-group preferences for telehealth consult modality in privately paying patients, patients who both identify as First Nations individuals and those who do not, patients aged 50–60, who did not require an interpreter, for the general population, and for the female population. We also found significant within-group preferences for in-person consult modality for patients who require an interpreter and for patients younger than 30.

**Discussion:**

Using the Maximum Entropy IRL sequential behaviour model, our results agree with previous evidence that non-attendance can be reduced when telehealth is offered in outpatient clinics. Our results complement previous studies using non-sequential modelling methodologies. Our preference and NA prediction results may be useful to outpatient clinic administrators to tailor services to specific patient groups, such as scheduling text message consult reminders if a given patient is predicted to be more likely to NA.

## 1 Introduction

Hospital outpatient clinics serve an important role in the Australian healthcare system by diverting patients with regular ongoing health needs away from centralised hospital inpatient resources. However, these clinics can experience a high rate of patients missing scheduled consults, referred to as non-attendance (NA), with one study finding NA rates between a 5% and 39% (1). Non-attendance causes additional burden on the outpatient services due to clinician time and other resources being wasted, and it lengthens wait lists for patients (1). Telehealth, the delivery of health services remotely using digital technologies, is one promising approach to accommodate patient needs while offering more flexibility in outpatient services (1, 2). However, there is limited evidence about whether or not offering telehealth consults as an option can change NA rates, or about the preferences of hospital outpatients for telehealth compared to in-person consults (1, 3–5).

Previous studies looking at this effect have used descriptive statistics and health economic methodologies including logistic regression and Discrete Choice Experiments (DCEs) (1, 6). Here, we investigate the use of the machine learning technique called Inverse Reinforcement Learning (IRL) to analyse the same problem. IRL is a behaviour modelling technique that attempts to rationalize observed sequential decision making behaviour by assuming the decision making agent is acting near-optimally, and finding a *reward function* that explains the observed behaviour (7). Unlike DCEs or logistic regression, IRL models decision-making behaviour as sequential reward optimization, allowing for agents that are forward thinking and anticipate future events, rather than acting myopically. Using a dataset of patient demographics and time-series attendance behaviour at an outpatient clinic located in a large metropolitan hospital in Brisbane, Australia, we model patient demographics as predictors of NA; or, that is, the demographic features of patients correspond to specific IRL behaviour models that in turn predict non-attendance likelihoods—and patient preferences for consult modality (telehealth or in-person)—that is, the IRL reward function parameters are interpreted as relative observed preferences.

We model patient preferences with the popular Maximum Entropy behaviour model (one version of the IRL technique), allowing for the calculation of general population- and demographic-specific relative preferences for consult modality. To allow comparison of our results with other health economic methodologies, we derive expressions to convert the Maximum Entropy IRL behaviour model to odds ratios for patient attendance or non-attendance. Because our IRL models can be queried for demographic- and/or modality-specific NA likelihoods, our preference and NA prediction results may be useful to outpatient clinic administrators to tailor services to specific patient groups, such as scheduling text message consult reminders if a given patient is predicted to be more likely to NA.

## 2 Methods

### 2.1 Ethics approval

Ethics approval for this research was granted by the Queensland Government Metro South Health District Human Research Ethics Committee, approval number HREC/2018/QMS/48636.

### 2.2 Data collection and processing

Activity data from October 2015 to September 2018 along with non-identifiable population characteristics for patients at a mixed in-person/telehealth immunology outpatient clinic associated with the Princess Alexandra Hospital (PAH) in Brisbane, Australia were obtained. The data were extracted from the PAH scheduling database and provided as a long-form password protected Microsoft Excel file, with associated codebook describing the columns and data types. For each scheduled consult, the data included non-identifiable patient demographic information, as well as if the patient attended or failed to attend, and the consult outcome which could include one of the following options:

(a) Re-booking the patient for a follow-up consult in either telehealth or in-person modality,
(b) Discharging the patient or referring them to another management service, indicating that the patient’s health needs were adequately resolved from the perspective of the clinic,
(c) Admitting the patient as an inpatient at the hospital, indicating an increase in the severity of the patient’s condition and the need for closer health management, or
(d) Removal of the patient from the clinic roster due to non-attendance one or more consults.

The dataset demographic characteristics were explored prior to analysis (Table 1).

**Table 1:** Patient demographic characteristics (*N* = 1026).

Characteristic	*n* (%) [missing *n*]
Male	369 (35.96%)
Consultation is privately funded	8 (0.78%)
Patient requires interpreter	30 (3.08%) [53]
Patient identifies as First Nations individuals	30 (3.13%) [67]
Patient age when entering clinic treatment	
<30 years old	190 (18.52%)
30–39 years old	211 (20.56%)
40–49 years old	185 (18.03%)
50–59 years old	179 (17.45%)
60–69 years old	143 (13.94%)
≥70 years old	118 (11.50%)

The raw data contained 6,131 consult lines corresponding to 1,790 unique patient interactions within the clinic during the collection time. From the raw data, we excluded 764 partially captured patient interactions that begun before the data capture window, leaving a total of 1,026 patient interactions for IRL modelling. Each patient interaction with the clinic consisted of between 1 and 13 scheduled consults, with the interaction lengths right-skewed (median patient interaction duration of two scheduled consults and a mean of 2.55 scheduled consults). Telehealth in this article refers specifically to videoconference calls and does not include any other technology modalities.

### 2.3 Maximum entropy Inverse Reinforcement Learning

We used IRL to model outpatient preferences with the Maximum Entropy (MaxEnt) behaviour model whose validation has been published elsewhere (8–10). IRL elicits observed preferences from a decision-making agent in an environment by finding a *reward function* which makes the observed behaviour appear optimal.

This is typically done in the context of a discrete-time Markov Decision Process (MDP), in which an agent observes the present state, takes an action, receives a scalar reward, then transitions to the next state. Specifically, we define a set of states s∈S that characterize the environment (a subset of which may terminate the MDP episode), and a set of actions that the agent can take a∈A. The MDP reward function (which is unknown but discovered using an IRL algorithm) provides a scalar reward signal when an action *a* is taken at state *s* r(s,a):S×A→R. A transition function describes the dynamics of the MDP as a probability distribution $T(s,a,s^{\prime })=p(s^{\prime }|\;s,a)$, and a probabilistic mapping from states to action distributions is referred to as a policy π(s,a)=p(a|s). We assume the observed agent is acting optimally, that is, they execute a policy $\pi^{*}$ (Equation 1) which maximizes their time-discounted expected reward,

(1) $$\pi^{*}=\mathrm{argma}x_{\pi }\;E\left[\overset{\infty }{\underset{t=0}{\sum }}\gamma^{t}\;r_{t}|p_{0}(s),\;T\right],$$

where $p_{0}(s)$ is a distribution over agent-starting states, and γ is a model hyper-parameter called the discount factor, which trades-off between near-term and future potential reward.

IRL elicits observed behaviour preferences from demonstration data $D=\{\tau_{i}{\}}_{i=1}^{N}$, where $\tau_{i}=(s_{0},a_{0},s_{1},a_{1},\ldots ,s_{L})$ denotes a length *L* state-action *trajectory* through the MDP (note that the scalar reward values received by the agent are not observed). To do this, IRL assumes a behaviour model, that is, a class of potential policies π∈Π and reward functions r(s,a)∈R. A popular choice is the maximum entropy (MaxEnt) behaviour model with a linear reward function, which assumes the policy takes the form

(2) $$\pi_{\theta }(\tau )\propto T(\tau )e^{r_{\theta }(\tau )},$$

where $T(\tau )=\underset{t}{\prod }T(s_{t},a_{t},s_{t+1})$ and $r_{\theta }(\tau )=\underset{t}{\sum }r_{\theta }(s_{t},a_{t})$, and the reward takes the parametric from

(3) $$r_{\theta }(s,a)=\theta^{⊤}\phi (s,a),$$

and ϕ(s,a) is a feature function. The normalizing constant for the MaxEnt trajectory distribution (Equation 2) is known as the *partition function* and can be efficiently computed with various inference algorithms (8, 9, 11), which is one reason for the popularity of the MaxEnt IRL framework.

The process of eliciting preferences using (Equation 3) MaxEnt IRL consists of defining the terms of the MDP (apart from the reward function), collecting a dataset of demonstration trajectories, then using optimization to search for the Maximum Likelihood Estimate (MLE) of the linear reward function parameter θ given the demonstration data. The reward function parameter can then be interpreted as a set of weights for the features in the feature function. Assuming a fixed feature function, the weights can also be interpreted as relative preferences for different state-action features in the environment.

### 2.4 Clinic MDP specification

**Figure 1:**
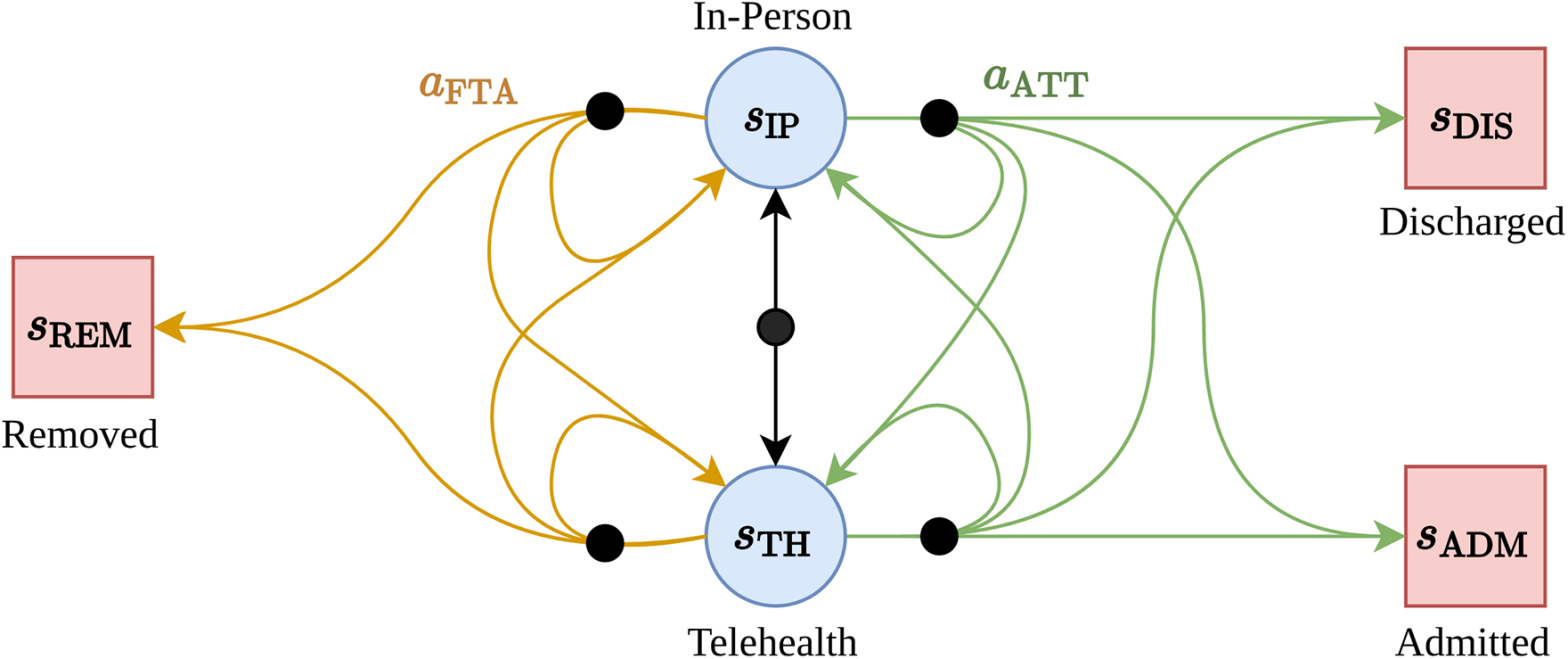
The MDP structure for the immunology outpatient clinic. Terminal states are shown as red boxes, regular states as blue circles. Black nodes indicate that the subsequent state is selected stochastically by the environment.

We modelled the patient interactions with the outpatient clinic as an MDP containing five states and two actions (Figure 1).

The states are: $s_{IP}$—the patient has a scheduled upcoming in-person consultation, $s_{TH}$—the patient has a scheduled upcoming telehealth consultation, $s_{DIS}$—(terminal state) the patient has been discharged from the clinic roster (*e.g.,* due to an improvement in their health condition), $s_{REM}$—(terminal state) the patient has been removed from the clinic roster (*e.g.,* due to repeated non-attendance), and $s_{ADM}$—(terminal state) the patient has been removed from the clinic roster due to admission as a hospital in-patient (*e.g.,* due to declining health condition). The actions available to the agent are: $a_{ATT}$—the patient attends the upcoming telehealth or in-person appointment, and $a_{NA}$—the patient does not attend the upcoming telehealth or in-person appointment. This MDP specification aligned with the data we collected—that is, patients experience a sequence of scheduled appointments with the clinic, and have control over weather or not they attend or do not attend each appointment. On the other hand, the clinic (the MDP “environment”) is responsible for determining if a patient is initially or subsequently (re-)booked for a telehealth or in-person appointment, discharged from the service, or admitted to the hospital.

The transition dynamics was estimated by clinic subject-matter experts, and the resulting model is shown in Table 2.

**Table 2:** Estimated parameters for the MDP transition dynamics.

Parameter	Description	Estimated value
$p_{IP}$	Probability a patient starts with an in-person consult	0.95
$p_{N}$	Probability of exiting the service after not attending a consult.	0.55
$p_{A}$	Probability of exiting the service after attending a consult.	0.15
d	Proportion of patients that attend a consult and leave the service by discharge rather than hospitalisation.	65%
$\sigma_{A}$	Odds of switching service modality after attending a consult.	1 in 1,000
$\sigma_{N}$	Odds of switching service modality after not attending a consult.	1 in 10,000

As a feature function, we opted for a state indicator vector—that is, a vector of zeros, with a single 1 entry corresponding to the most recently selected state and action, $\phi (s,a)=(1_{s=s_{1}},\ldots ,1_{s=s_{n}})$, where *n* is the number of states, and $1_{s=s_{i}}$ is an indicator function returning 1 if and only if $s=s_{i}$, or returning 0 otherwise. We model our patients as far-sighted (the opposite of myopic) by selecting a discount factor close to one (γ=0.999).

### 2.5 Eliciting outpatient appointment preferences

The MaxEnt IRL reward function parameters θ for the target population were estimated by maximizing the likelihood of the demonstration data using full-batch gradient descent with the L-BFGS optimizer, constraining the parameter values to lie in the set $\theta \in [-1,+1{]}^{\left|S\right|}$ to make interpretation of the weights simpler. The gradient and objective terms were computed using the exact MaxEnt IRL inference algorithm previously published by Snoswell, Singh, and Ye (8). Python 3.6.9 and the scipy library (12) were used for all numerical calculations. To estimate demographic group specific preferences, the data were partitioned by demographic groups, and group-specific reward parameters calculated in the same manner as just described. After optimization, the learned reward parameters were interpreted as relative observed preferences for different states within the MDP, allowing comparison with the relative stated preferences elicited in parallel work using a tailored Discrete Choice Experiment in a patient survey (6).

To measure the uncertainty for the reward parameters, we compute the 95% boostrap confidence intervals centered around the reward parameter estimates (13).

### 2.6 Predicting outpatient non-attendance

After learning the reward function parameters for each patient demographic group, the learned reward function values were used to predict outpatient attendance or non-attendance. Such a problem can be readily solved by treating the maximum entropy distribution induced by the learned reward parameters (Equation 2) as a stochastic policy which encodes a preference over alternate futures through the MDP, and querying this policy for the relative probability of a patient attending or failing to attend.

The standard health-economic tool for reporting such predictions, such as clinic attendance or non-attendance, is the “Odds Ratio” (OR), defined as the odds of an outcome occurring in the presence of some intervention divided by the odds of that outcome in the absence of the intervention. For instance, the OR for non-attendance if a consult is via telehealth (instead of in-person) can be computed as

(4) $$OR_{\mathrm{NA}\;|\;\mathrm{Telehealth}}=\frac{\;p(\mathrm{NA}\;|\;\mathrm{Telehealth})}{\;p(\mathrm{NA}\;|\;\mathrm{In}\text{-}\mathrm{Person})}$$

We compute the odds ratio in (Equation 4) as

(5) $$OR_{\mathrm{NA}\;|\;\mathrm{Telehealth}}=\frac{\sum_{t=1}^{L-1}p_{\theta ,t}(s_{\mathrm{TH}},a_{\mathrm{NA}})/\;p_{\theta ,t}(s_{\mathrm{TH}})}{\sum_{t=1}^{L-1}p_{\theta ,t}(s_{\mathrm{IP}},a_{\mathrm{NA}})/\;p_{\theta ,t}(s_{\mathrm{IP}})},$$

where *L* is an upper time-horizon (the maximum number of steps into the future the simulated agent plans when making decisions), and where $p_{\theta ,t}(s,a)=p(s_{t}=s,a_{t}=a|\tau ∼p_{\theta }(\tau ))$ and $p_{\theta ,t}(s)=p(s_{t}=s|\tau ∼p_{\theta }(\tau ))$ are the state-action and state marginal counts induced by the reward parameter θ, which can be exactly and efficiently computed using the inference algorithm described in Snoswell, Singh, and Ye (8).

On the other hand, if $p(a_{\mathrm{NA}}\;|\;\theta_{B})$ is the probability of non-attendance in the presence of a demographic trait *B*, and $p(a_{\mathrm{NA}}\;|\;\theta_{\neg B})$ is the probability of non-attendance in the absence of that trait, then we can compute the trait-dependent OR of non-attendance with the expression

(6) $$OR_{\mathrm{NA}\;|\;B}=\frac{\;p(a_{\mathrm{NA}}\;|\;\theta_{B})}{\;p(a_{\mathrm{NA}}\;|\;\theta_{\neg B})}=\;\frac{\sum_{s\in S}\sum_{t=1}^{L-1}p_{\theta_{B},t}(s,a_{\mathrm{NA}})/\;p_{\theta_{B},t}(s)}{\sum_{s^{\prime }\in S}\sum_{t=1}^{L-1}p_{\theta_{\neg B},t}(s^{\prime },a_{\mathrm{NA}})/\;p_{\theta_{\neg B},t}(s^{\prime })}$$

We computed ORs from the MaxEnt model for consult modality (using Equation 5) and for the categorical patient demographic variables (using Equation 6). To measure the uncertainty of these estimates, we used bootstrap re-sampling with replacement on each data set/subset to compute a mean ORs and symmetric 95% confidence intervals.

## 3 Results

### 3.1 Modelling outpatient appointment preferences

The computed patient reward parameters with 95% confidence intervals are shown in (Table 3). The primary terms of interest are the relative strength of the preferences for in-person consults $\theta_{\mathrm{IP}}$ vs. telehealth consults $\theta_{\mathrm{TH}}$. To investigate these terms, we selected a null hypothesis that the difference between telehealth and in-person preference was equal to zero:

$$H_{0}:\theta_{\mathrm{TH}}-\theta_{\mathrm{IP}}=0$$

$$H_{1}:|\theta_{\mathrm{TH}}-\theta_{\mathrm{IP}}|>0$$

**Table 3:** Computed reward parameters (mean and 95% CI over 100 bootstrap re-samples) for patient demographic groups.

Group	$\theta_{\mathrm{IP}}$	$\theta_{\mathrm{TH}}$	$\theta_{\mathrm{DIS}}$	$\theta_{\mathrm{ADM}}$	$\theta_{\mathrm{REM}}$
All	−0.76 ± 0.01	**−0.71 ± 0.01**	0.95 ± 0.02	−0.63 ± 0.09	−0.98 ± 0.02
Male	−0.80 ± 0.01	−0.77 ± 0.03	0.94 ± 0.04	−0.72 ± 0.10	−0.85 ± 0.05
Female	−0.74 ± 0.01	**−0.69 ± 0.02**	0.96 ± 0.02	−0.55 ± 0.10	−1.00 ± 0.00
Public	−0.76 ± 0.01	−0.75 ± 0.02	0.97 ± 0.02	−0.66 ± 0.08	−0.99 ± 0.01
Private	−1.00 ± 0.00	**−0.29 ± 0.04**	0.10 ± 0.35	−1.00 ± 0.00	−0.73 ± 0.15
Interpreter required	**−0.77 ± 0.03**	−1.00 ± 0.00	0.56 ± 0.20	−1.00 ± 0.00	−0.74 ± 0.12
No interpreter required	−0.75 ± 0.01	**−0.69 ± 0.02**	0.98 ± 0.02	−0.53 ± 0.10	−1.00 ± 0.00
First Nations individuals	−0.86 ± 0.03	**−0.63 ± 0.06**	0.29 ± 0.25	−0.22 ± 0.26	−0.19 ± 0.15
Non- First Nations individuals	−0.74 ± 0.01	**−0.72 ± 0.02**	0.98 ± 0.02	−0.58 ± 0.10	−1.00 ± 0.00
Age <30	**−0.76 ± 0.01**	−0.83 ± 0.03	0.76 ± 0.07	−0.89 ± 0.09	−0.72 ± 0.06
Age 30s	−0.78 ± 0.01	−0.78 ± 0.04	0.42 ± 0.09	0.01 ± 0.14	−0.78 ± 0.06
Age 40s	−0.74 ± 0.02	−0.72 ± 0.05	0.97 ± 0.03	−0.70 ± 0.13	−0.92 ± 0.04
Age 50s	−0.78 ± 0.01	**−0.70 ± 0.03**	0.98 ± 0.02	−0.85 ± 0.09	−1.00 ± 0.01
Age 60s	−0.74 ± 0.02	**−0.66 ± 0.04**	1.00 ± 0.01	−0.69 ± 0.14	−1.00 ± 0.00
Age ≥70	−0.80 ± 0.02	−0.75 ± 0.04	0.99 ± 0.02	−0.34 ± 0.18	−1.00 ± 0.00

Where a statistically significant (α=0.05) within-group preference for in-person or telehealth consult type exists, the preferred option is highlighted in bold.

Performing bootstrap hypothesis testing with 100 re-samples (14), we found statistically significant (α=0.05) within-group preferences for telehealth in the following groups (ranked from weakest to strongest effect): for privately paying patients ($\theta_{\mathrm{TH}}-\theta_{\mathrm{IP}}=0.71\pm 0.04$), for patients who identify as either First Nations individuals (0.23±0.07) or those who do not (0.03±0.02), for patients aged in their 50s (0.08±0.03) or 60s (0.07±0.04), for those who indicated they did not require an interpreter for their consult (0.06±0.01), for the general population (0.05±0.01), and for the female population (0.05±0.02).

On the other hand, we found statistically significant (α=0.05) within-group preferences for in-person consults for patients who routinely require an interpreter for their consults ($\theta_{\mathrm{IP}}-\theta_{\mathrm{TH}}=0.23\pm 0.03$) and for patients younger than 30 (0.07±0.04).

As expected, in all cases, the telehealth and in person reward weights were negative, which suggests that patients are motivated to reach a terminal state (and cease interaction with the outpatient clinic) promptly. That is, patients want to exit the health system by either resolving their condition or ceasing contact with the clinic through referral sooner rather than later.

Encouragingly, some of the learned preferences here match intuitive expectations. For instance, a preference for in-person consults for patients requiring an interpreter makes intuitive sense due to the potential difficulties of establishing a remote connection to the hospital telehealth system without an interpreter physically present with the patient. Likewise, the fact that patients identifying as First Nations individuals appear to prefer telehealth, as demonstrated by their strong preference difference between in-person and telehealth consults, is likely due to the potential that such patients may be physically located in remote rural communities, or may desire to have family present. These factors may also drive the preferences for those who do not identify as First Nations individuals, however the difference between their preferences for in-person or telehealth was smaller by a factor of ten (a difference in the in-person and telehealth reward parameters of 0.23 for First Nations individuals vs. 0.02 for those that do not identify as First Nations). Encouragingly, the general trend of a baseline preference for telehealth consult was also reflected in the DCE results reported elsewhere (6).

### 3.2 Predicting outpatient non-attendance

We computed non-attendance odds ratios for telehealth vs. in-person consultation modality and for patient demographic characteristics (Table 4).

**Table 4:** Non-attendance odds ratios for consult and patient characteristics.

Characteristic	Variables	Odds ratio	95% CI	Interpretation
Appointment modality	Telehealth/in person	1.0231	1.0200–1.0262	Marginal increased NA predicted for telehealth consults.
Sex	Female/Male	0.9965	0.9907–1.0024	Inconclusive.
Source of funding	Private/Public	1.1447	1.1310–1.1584	Increased NA predicted for patients that pay privately.
Interpreter required	Interpreter required/No interpreter required	1.0616	1.0469–1.0763	Marginal increased NA predicted for patients that require an interpreter.
First Nations individuals identification	First Nations individuals/Non- First Nations individuals	1.1927	1.1720–1.2134	Increased NA predicted for patients that identify as First Nations individuals

Values are mean and 95% CI over 100 bootstrap re-samples.

These results indicate that patients are marginally more likely to NA if a consult is offered via telehealth or if a patient requires an interpreter, and that patients are more likely to NA if they pay privately for their consult, or identify as First Nations individuals. The Odds ratio for patient sex is non-conclusive (confidence interval ranges both above and below 1.0).

## 4 Discussion

We have demonstrated the use of the MaxEnt behaviour model for modelling the preferences of hospital outpatients (via reward learning) as well as for predicting likely actions (via imitation learning). Our results, when compared with related studies on the same data (1), and on a similar population (6), suggest that IRL may be a promising methodology for health economic modelling, alongside logistic regression and DCEs. The directions of our statistically significant trait-dependent ORs (Equation 6) (*<*1 or *>*1) match those from a logistic regression on the same data with the exception of the OR Equation (5) for the general population, and for consult funding source (1). The general population OR is marginally above 1.0, indicating a very small increase in the odds of NA when a consult is offered over telehealth, which does not match previous findings (1). The consult funding source OR is positive, which is unexpected, given that it would seem that patients have a vested interest in attending a consult if they (or their health fund) are paying out-of-pocket for an consult, however this is likely due to the small sample size for the privately paying sub-population.

Some of the key results around non-attendance likelihood and preference have been highlighted previously by other research, providing external validity to the modelled results. For instance, higher non-attendance rates among First Nations individuals has been demonstrated for both general practice and medical imaging appointments (15, 16). The finding that individuals under the age of 30 prefer in-person consults compared to telehealth is unusual and should be explored in future research. Our results around the marginal preference for telehealth and its ability to affect non-attendance rates aligns with literature published prior to the COVID-19 pandemic, as the COVID-19 pandemic also saw a instantaneous shift in how individuals access healthcare (1, 17–19). Rerunning this model with post-pandemic data may be an interesting exercise to further explore this relationship.

One limitation of our approach is that the MDP specification is in discrete time—that is, there is no notion of “waiting time” or elapsed duration between appointments—agents in the MDP simply move from one appointment to the next. Prior research has studied the effect of indirect waiting time on no-show probability (20), however our formulation does not capture this variable. An alternate MDP specification could either use a continuous-time framing, or additional “waiting” states to capture the elapsed real-world time between appointments, which might then allow studying this variable with the IRL formulation.

A weakness of the approach to preference elicitation used here is that the bootstrap estimates do not allow for testing the statistical significance or non-significance of learned preferences *between*-groups. This is especially important to keep in mind, given that some of the demographic groups had very few available demonstration trajectories (*e.g.,* privately paying patients had only *n *= 8 data points), recommending caution when interpreting any apparent between-group differences. Future work can be done to validate the model with additional real world data by studying e.g., a larger population but for a similar outpatient clinic model or treatment scenario, as well as looking a treatment scenarios that involve longer trajectories (more patient-clinic interactions over time). In the future, theoretical work is also needed to complement IRL modelling approaches with a richer set of statistical significance testing approaches.

For all variables, the ORs suggest a relatively small effect size compared to the logistic regression results (*e.g.,* MaxEnt OR's ranging from 0.9965–1.1927 vs. logistic regression OR's ranging from 0*.*32–4*.*66). This suggests an interesting possibility when we consider that logistic regression is essentially a predictive model that collapses the data to a single time-step, whereas IRL considers the impact of sequential decisions over time. As such, the results here suggest that our predictions of NA (or attendance) become weaker (smaller effect size) as we generalise our modelling approach from treating patients as making isolated single time-step decisions to modelling patient behaviour as rational goal directed decision making over time.

Another relevant factor to consider is the assumptions implicit in the chosen behaviour model. For instance, a DCE models patients as myopic (making a single-timestep decision without any consideration of future possible outcomes) (21). IRL methods, as a form of agent-based modelling, relax this restrictive assumption but come with their own assumptions. For instance, the maximum entropy behaviour model we have used here assumes that decision making agents care about the trajectory-level feature moments, however other behaviour models (such as ML-IRL (22) or Σ-GIRL (24) could also be used, and would bring their own modelling limitations and/or hyper-parameters as well.

It remains an important IRL research problem how to estimate dynamics models in a data-driven fashion without requiring subject matter expert input (23–27), as well as the development of more rigorous statistical significance tests for learned reward and policy parameters. This poses a significant challenge due to the non-trivial mathematical operations required (and assumptions entailed) in learning rewards and policies. Here, we have used the bootstrap re-sampling method (14) to provide one measure of uncertainty, however this method has known limitations (13, 28).

Our experiments modelling patient preferences and behaviors from real-world medical data highlighted how IRL methods can provide similar insights alongside more traditional health economic analyses. This work pushes forward the theory and practice of IRL on multiple fronts. By developing theory and algorithms for efficient and exact learning of MaxEnt IRL reward parameters, by extending these algorithms to multiple new problem classes, and by demonstrating the techniques required to apply these methods to real-world problems, we hope that we can inspire new interest in the MaxEnt IRL model, and also in IRL more generally as a methodology for understanding behaviour within medical research.

## Data Availability

The datasets presented in this article are not readily available because specific ethics and governance approvals are required to access the patient data used for this analysis. Requests to access the datasets should be directed to Dr. Centaine L. Snoswell c.snoswell@uq.edu.au.
